# Preparation of Calcium–Binding Peptides Derived from Mackerel (*Scomber japonicus*) Protein and Structural Characterization and Stability Analysis of Its Calcium Complexes

**DOI:** 10.3390/foods13111652

**Published:** 2024-05-25

**Authors:** Pengbo Cui, Jianqin Liang, Tianyu Cheng, Jianyou Zhang

**Affiliations:** 1 Zhejiang Key Laboratory of Green, Low-Carbon and Efficient Development of Marine Fishery Resources, College of Food Science and Technology, Zhejiang University of Technology, Hangzhou 310014, China; liangjianqin@zjut.edu.cn (J.L.); 2112126071@zjut.edu.cn (T.C.); zhjianyou@zjut.edu.cn (J.Z.); 2 National Engineering Research Center of Seafood, School of Food Science and Technology, Dalian Polytechnic University, Dalian 116034, China

**Keywords:** mackerel, calcium-binding peptide, characterization, stability analysis

## Abstract

The purpose of this study was to prepare mackerel peptides (MPs) with calcium-binding capacity through an enzyme method and to investigate the potential role they play in improving the bioavailability of calcium in vitro. The calcium-binding capacity, degree of hydrolysis (DH), molecular weight (MW), and charge distribution changes with the enzymolysis time of MPs were measured. The structural characterization of mackerel peptide–calcium (MP–calcium) complexes was performed using spectroscopy and morphology analysis. The results showed that the maximum calcium-binding capacity of the obtained MPs was 120.95 mg/g when alcalase was used for 3 h, with a DH of 15.45%. Moreover, with an increase in hydrolysis time, the MW of the MPs decreased, and the negative charge increased. The carboxyl and amino groups in aspartic (Asp) and glutamate (Glu) of the MPs may act as calcium-binding sites, which are further assembled into compact nanoscale spherical complexes with calcium ions through intermolecular interactions. Furthermore, even under the influence of oxalic acid, MP–calcium complexes maintained a certain solubility. This study provides a basis for developing new calcium supplements and efficiently utilizing the mackerel protein resource.

## 1. Introduction

Calcium is an essential nutrient for the human body, making up 1.5–2.2% of total body weight [1], and participates in many biological functions, such as bone growth, muscle contraction, nerve conduction, and cell phagocytosis [2,3,4,5]. However, the calcium intake in more than 95% of Chinese adults is insufficient, which can lead to many diseases, such as osteoporosis, rickets, and hypertension [6]. Additionally, food components, such as phytic, oxalic, and phosphoric acids, readily precipitate ingested calcium in the intestine, inhibiting the intestinal absorption of calcium ions and further decreasing the calcium bioavailability [6]. Therefore, the development of easily absorbable calcium supplements is crucial for mitigating deficiency.

At present, the main calcium supplements include inorganic calcium, organic calcium, amino-acid-chelated calcium, and peptide–calcium complexes [7]. The disadvantage of ionized calcium is that calcium precipitation can easily occur in the intestinal environment, and amino-acid-chelated calcium is expensive [8,9]. Compared to free amino acids (AAs), peptides offer advantages such as a high absorption rate, low energy consumption, and a lack of carrier saturation [10]. Various studies have revealed that peptides rich in Asp and Glu can bind with calcium and further assemble into soluble and stable nanocomposites to promote calcium absorption effectively in vivo or in vitro [11,12]. This is the current research focus in the field of calcium supplements. Enzymatic hydrolysis is widely applied in the preparation of calcium-binding peptides [13]. However, the relationship between the calcium-binding capacity of peptides and the type of enzyme, degree of hydrolysis (DH), molecular weight (MW), and charge state of the hydrolysates still needs further clarification.

Mackerel, *Scomber japonicus*, is widely distributed throughout the North Pacific Ocean and is an important fishery resource worldwide, with an annual catch of approximately 1.5 million metric tons [14,15]. In a previous study, we found that defatted mackerel meat is rich in Asp and Glu, which are potential sources of calcium-binding peptides. However, the calcium-binding properties of mackerel peptides (MPs) have not been investigated. Therefore, we hypothesized that MPs combined with calcium could provide a low-cost and highly bioavailable calcium supplement. The objective of this study was to determine the best enzyme for the preparation of MPs with high calcium-binding capacity and to study the calcium-binding capacity and how DH, MW, and charge distribution change with enzymolysis time. Additionally, the structure of the MP–calcium complexes was characterized via spectroscopic and morphological analyses. Finally, the gastrointestinal (GI) digestion stability of MP–calcium complexes in the presence of dietary factor oxalic acid was evaluated through an in vitro GI digestion model. The findings of this study may provide a basis for developing new calcium supplements and efficiently utilizing mackerel protein resources.

## 2. Materials and Methods

### 2.1. Materials

Mackerels (*S. japonicus*) were obtained from a local market (Deqing, Huzhou, China). Alcalase (200 u/mg), flavourzyme (20,000 u/mg), and papain (800 u/mg) were purchased from Yuanye Biotechnology Co., Ltd. (Shanghai, China). Trypsin (250 u/mg) was obtained from Macklin (Shanghai, China). Other chemicals and reagents used in the present study were of analytical grade and commercially available.

### 2.2. Preparation of MPs

Mackerel meat was degreased at 50 °C for 6 h, filtered, and crushed after drying at room temperature in a fume hood for 12 h [16]. The degreased mackerel powder was mixed with deionized water at a ratio of 1:50 (*w*/*v*). The mixture was hydrolyzed with trypsin or flavourzyme at 37 °C and pH 8.0 at a concentration of 3000 U/g protein. At 50 °C, alcalase (pH 8.5) or papain (pH 7.0) was used for enzymatic hydrolysis under optimal pH conditions at a concentration of 3000 U/g protein. After enzymatic hydrolysis for 3 h, the enzyme was heated for 10 min at 100 °C to render it inactive. After centrifuging the suspension for 20 min at 10,000× *g*, 4 °C, the precipitate was disposed of. After being freeze-dried and labeled as MPs, the supernatant was stored at −20 °C for future study.

### 2.3. Determination of DH

The measurement of DH was measured using the pH-stat method, with specific operational steps following the previous studies of Sun et al. [16].

### 2.4. Analysis of MP Characterization

#### 2.4.1. Calcium-Binding Capacity Assay

The determination of calcium-binding capacity, which is defined as the calcium (mg) content bound to the peptide (g) after the binding reaction, was performed using the EDTA titration method following our previous study [17]. The formula for the calculation is as follows:

calcium (%) = 40 CV/M,

where C is the concentration of the EDTA standard (0.02 M) solution, V is the amount of EDTA standard solution consumed (mL), and M is the mass (g) of the sample.

#### 2.4.2. Determination of MW Distribution

The MW distribution of MPs for different hydrolysis times was determined via HPLC following Zhang et al. [1]. The chromatography was performed on an XBridge Protein BEH 125A SEC (3.5 μm, 7.8 × 300 mm) column (Waters Corp., Milford, MA, USA) with mobile phase acetonitrile/water/trifluoroacetic acid (40:60:0.1, *V*/*V*/*V*), UV detection wavelength 220 nm, flow rate 0.4 mL/min, column temperature 30 °C, and sample size 10 μL. Cytochrome C (12,384 Da), aprotinin (6511.51 Da), bacitracin (1422.69 Da), L-oxidized glutathione (612.63 Da), and hydroxyproline (131.13 Da) were used as standard materials to construct relative MW standard curves. The MPs were then prepared as a solution with a final concentration of 1 mg/mL and passed through a 0.22 μm filter membrane; the sample was measured on a computer. The MW distribution of MPs was calculated using the standard curve equation.

#### 2.4.3. Zeta Potential Measurement

The zeta potential of MPs was determined according to the method described by Sun et al. [16], and analyzed using Malvern Nano ZS Zetasizer based on dynamic light scattering. MPs were dissolved in distilled water at a concentration of 1 mg/mL. All measurements were performed at 25 °C, and the results were reported as the average of three readings.

#### 2.4.4. Analysis of Amino Acid (AA) Composition

The AA composition analysis was performed using a Biochrom30+ AA analyzer, as described in our previous study [16]. Before derivatization with 2,4-dinitrofluorobenzene, all AA residues were determined after hydrolysis with 6 M HCl at 110 °C for 24 h.

### 2.5. Preparation of MP–Calcium Complexes

Lyophilized MPs were dissolved in deionized water at a concentration of 50 mg/mL, and CaCl_2_ was subsequently added to obtain a peptide/Ca mass ratio of 3:1. The binding reaction was conducted at 50 °C and pH 8.5 for 30 min. Then, the free calcium and sodium chloride were eliminated by adding 85% ethanol to the reactant. After centrifugation at 8000× *g* for 10 min, the precipitate was collected, freeze-dried, and labeled as MP–calcium complexes [18].

### 2.6. Structural Characterization of MPs and MP–Calcium Complexes

#### 2.6.1. Fluorescence Spectroscopy

The fluorescence spectra of the samples were recorded over emission wavelengths ranging from 320 to 500 nm, at an excitation wavelength of 280 nm, using a Hitachi F-2700 fluorescence spectrophotometer (Hitachi Co., Tokyo, Japan).

#### 2.6.2. Fourier Transform Infrared (FTIR) Measurement

The FTIR spectroscopic analysis of the samples was performed using an FT-IR spectrometer (Bruker, Ettlingen, Germany) over a wavenumber range between 4000 and 400 cm^−1^ at a resolution of 4 cm^−1^.

#### 2.6.3. Atomic Force Microscopy (AFM)

AFM images were obtained by AFM (Bruker Dimension Icon, Bruker, Bremen, Germany), according to the method described by Zhang et al. [19] with some modifications. First, 2 mg/mL of MPs and MP–calcium complexes were dissolved in deionized water. Then, 20 μL of the mixture was deposited on silicon wafers, spread, and air-dried naturally at room temperature. These images were obtained in true noncontact mode.

#### 2.6.4. Scanning Electron Microscopy (SEM) and Energy Dispersion Spectrum (EDS)

SEM and EDS analyses were conducted according to the method described by Qu et al. [20], with some modifications; the measurement was performed on Scanning Electron Microscopy (Sigma 300, Carl Zeiss AG, Oberkochen, Germany). Gold-plated MPs and MP–calcium powder were attached to the SEM device. The samples were observed and captured at an accelerated voltage of 3 kV. The element composition of the sample was analyzed through point scanning using the EDS method.

### 2.7. Bio-Accessibility Measurement of the MP–Calcium Complexes

#### 2.7.1. Calcium Solubility Assay in Different Temperature Conditions

The stability tests of the MP–calcium complexes at different temperatures were evaluated according to the methods of Qu et al. [21] with some modifications. MP–calcium complexes (2 mg/mL) were incubated at 40, 50, 60, 70, and 80 °C for 2 h and then centrifuged at 8000× *g* for 10 min to determine the calcium content in the supernatant.

#### 2.7.2. Calcium Solubility Assay under Simulated GI Digestion

According to the method of O’Loughlin et al. [22] and Sun et al. [18], an in vitro simulated GI digestion model was used to study the calcium solubility of MP–calcium complexes during the digestion process. First, pepsin (40 mg) was dissolved in 1 mL of 0.1 N HCl to prepare a simulated gastric digestion solution. A simulated intestinal digestive solution was prepared by dissolving 20 mg of trypsin and 120 mg of sodium taurocholate in 10 mL of 0.1 M NaHCO_3_. Then, 0.2 g MP–calcium complexes and 0.2 g CaCl_2_ were dissolved in 60 mL of ultrapure water and incubated at 37 °C for 30 min. The pH was adjusted to 2.0, 0.05 mL of simulated gastric digestion solution was added, and gastric digestion was simulated at 37 °C. At digestion intervals of 0, 10, 30, 60, and 90 min, 4 mL of the solution was taken out, and the enzyme was sterilized in a water bath at 100 °C for 5 min. The mixture was centrifuged at 10,000× *g* for 10 min, and the calcium content of the supernatant was measured. After 90 min of simulated gastric digestion, the pH was adjusted to 7.5, 0.17 mL of simulated intestinal digestion liquid was added, and the simulated intestinal digestion was continued at 37 °C. At digestion intervals of 0, 5, 10, 30, 60, 90, and 150 min, a small amount of liquid was taken out and heated for 20 min in an 80 °C water bath to inactivate the enzyme. Each sample was centrifugated at 10,000× *g* for 10 min, the supernatant was removed, and its calcium content was determined. At the same time, according to the methods of Jiang et al. [23] and Hu et al. [24], the effect of oxalic acid on calcium absorption was studied. MP–calcium or CaCl_2_ was dissolved in deionized water, and 5 mL, 2% oxalic acid was added. As mentioned above, the mixture underwent a two-stage GI process. The sample was centrifuged at 4 °C, 10,000× *g* for 10 min, and the supernatant was collected for the determination of calcium content.

### 2.8. Statistical Analysis

All experiments were repeated thrice. SPSS 18.0 was used for one-way analysis of variance of all data. The mean value was compared using the least significant test, and the confidence level was set as *p* < 0.05. All diagrams were plotted using Origin 2019 software.

## 3. Results and Discussion

### 3.1. Characterization of the MPs

#### 3.1.1. Effect of Proteases on DH and Calcium-Binding Capacity of MPs

**Figure 1:**
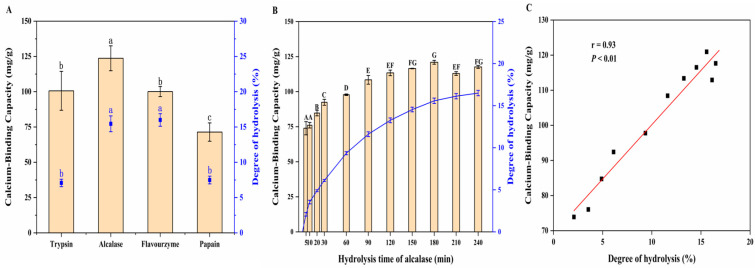
Effects of enzyme type and DH on calcium-binding capacity. (**A**) Effects of enzyme type on MPs’ calcium-binding capacity and DH. (**B**) Effects of different hydrolysis times on calcium-binding capacity of the MPs obtained with alcalase. (**C**) Correlation of calcium-binding capacity of the MPs with DH. Different letters indicate significant differences among groups (*p* < 0.05).

Enzymatic hydrolysis is a useful technique for modifying proteins, especially for improving protein digestibility and producing bioactive peptides. The reaction conditions were mild, controllable, and did not produce toxic decomposition products, thus reducing allergic reactions [25,26]. Many researchers use enzymatic hydrolysis to prepare calcium binding peptides. We measured the DH and calcium-binding capacity of the MPs obtained using four different proteases (trypsin, alcalase, flavourzyme, and papain), as shown in Figure 1A. The DH of MPs was the highest for flavourzyme (16.00%) and the lowest for trypsin (7.08%). The DH of MPs prepared with alcalase was similar to that of MPs prepared with flavourzyme, reaching 15.45%, which was significantly higher than that of the enzymatic hydrolysates produced with trypsin and papain (7.49%). Alcalase contains a wide range of non-specific protease digestion sites and can recognize more AA sites [27], while papain has a specific choice of substrate. Flavourzyme exhibits both endonuclease and exopeptidase activities, which can improve the DH via the terminal hydrolysis of peptides [28]. Additionally, different MPs exhibited different calcium-binding capacities. MPs produced with alcalase showed the highest calcium-binding capacities, whereas those obtained with papain showed the lowest (*p* < 0.05). A similar result was reported previously; alcalase was more effective than flavourzyme and papain in producing metal-binding peptides from wheat germ proteins, with a DH of 15.61 ± 0.09% and metal-binding capacity of 69.62 ± 0.96% [29]. This finding could be attributed to the strong specificity of alcalase to aromatic, aliphatic, hydroxyl, and acidic residues, which can effectively break the peptide bond structure, thus releasing more Ca^2+^-binding sites [24]. Through a comprehensive analysis of the calcium-binding ability and DH of MPs prepared with four kinds of enzymatic hydrolysis, we found that alcalase could achieve better effects. Therefore, alcalase was selected for subsequent studies.

#### 3.1.2. Effect of Hydrolysis Time on Calcium-Binding Capacity of MPs

The calcium-binding capacity of the MPs obtained using alcalase after different hydrolysis times was studied, and the results are shown in Figure 1B. During the first 3 h of hydrolysis, the calcium-binding capacity increased with increasing hydrolysis time. When the enzymatic hydrolysis time was 3 h, the calcium-binding capacity was the highest, and 120.95 mg/g of calcium was present in the MP–calcium complexes. However, when the enzymatic hydrolysis time exceeded 3 h, the calcium-binding capacity decreased, indicating that the hydrolysis time significantly impacted the binding reaction of MPs with calcium. This result aligns with that of Wu et al. [30], in which the iron-binding activity of anchovy muscle protein peptides increased with increasing hydrolysis time and then decreased after more than 4 h. This may be because enzymatic hydrolysis produces calcium-binding peptides, whereas excessive hydrolysis reduces their activity. Additionally, a close correlation was observed between DH and calcium-binding capacity (r = 0.93, *p* < 0.01) (Figure 1C). These results indicate that DH affects the calcium-binding activity of MPs obtained using alcalase, and the calcium-binding ability was the highest when enzyme hydrolysis was run for 3 h.

#### 3.1.3. MW Distribution

**Figure 2:**
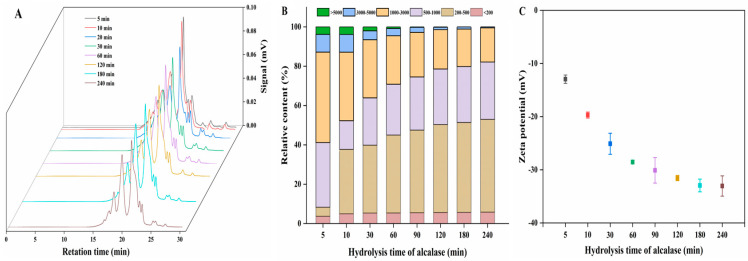
MW distribution and zeta potential profile of the MPs treated with alcalase at different hydrolysis times. (**A**) MW distribution profile of the MPs. (**B**) The relative content of different MW MPs obtained at different hydrolysis times. (**C**) The zeta potential values of the MPs at different hydrolysis times.

MW plays an important role in calcium-binding reactions. Peptides with low MW often exhibit high calcium-binding ability in the reactions; however, some peptides with higher MW also show good calcium-binding capacity [5,31]. To determine the effect of the MW of MPs on the calcium-binding activity, the MW distribution profile of MPs at different enzymatic hydrolysis times was measured (Figure 2A). The percentage content of each MW range was represented by the percentage area under the curve, as shown in Figure 2B. With an increase in enzymatic hydrolysis time, the relative proportion of fractions between 200 and 1000 Da increased significantly from 37.49% to 76.83% (*p* < 0.05), whereas the proportion of fractions larger than 1000 Da decreased significantly from 58.82% to 17.83% (*p* < 0.05). Additionally, with a decrease in MW, the calcium-binding capacity of the MPs increased significantly. According to the correlation analysis, a small proportion between 200 and 1000 Da was positively correlated with the calcium-binding activity (r = 0.97, *p* < 0.01). In contrast, there was a negative correlation between calcium-binding activity and component content >1000 Da (r = 0.97, *p* < 0.01). These results suggest that the MW of MPs decreased with the prolongation of enzymolysis time, and the calcium binding capacity of MPs was proportional to the low molecular content. Therefore, the MW of MPs was an important factor affecting the calcium-binding capacity, and the low-MW hydrolysate was favorable for calcium binding.

#### 3.1.4. Zeta Potential Analysis

Zeta potential measures the amount of charge on the particle surface in the dispersion system and indicates the stability of the system. This is also helpful for exploring the potential mechanism underlying electrostatic interactions between calcium ions and polypeptides [32]. Figure 2C shows the zeta potentials of MPs obtained at different hydrolysis times. With the extension of the hydrolysis time, the negative charge on the MPs gradually increased, possibly because the acidic AAs were gradually exposed, which may be calcium-binding sites. When hydrolyzed for 3 h, MPs carried a mostly negative charge, consistent with the time when the highest calcium-binding amount occurred. Additionally, the negative charge carried by the MPs obtained after different hydrolysis times was positively correlated with calcium-binding activity (r = 0.98, *p* < 0.01). Therefore, enzymatic hydrolysis increases the exposure of negatively charged components, which may serve as calcium-binding sites.

#### 3.1.5. Amino Acid Composition Analysis 

The AA compositions of the MPs and MP–calcium complexes are shown in Table 1. MPs are rich in various AAs essential for the human body, which can be used as high-quality AA supplements. Additionally, significant differences were observed in the AA compositions of MPs and MP–calcium complexes before and after the binding reaction, likely due to the dependency of metal ions binding on specific AAs [33,34]. After combining with Ca^2+^ to form MP–calcium complexes, the relative content of Asp + Asn and Glu + Gln significantly increased. This finding might be attributed to Asp and Glu being acidic AAs, and their residues provide sites for combining metal ions and peptides [35]. They can change the local charge density through the side-chain carboxylic acid groups and enhance the metal-binding ability of the ligand, facilitating the assembly of peptides and calcium [3]. We speculated that Asp and Glu on MPs might interact with calcium via intermolecular interactions.

### 3.2. Structural Characterization of MP–Calcium Complexes

#### 3.2.1. Fluorescence Spectroscopy

**Figure 3:**
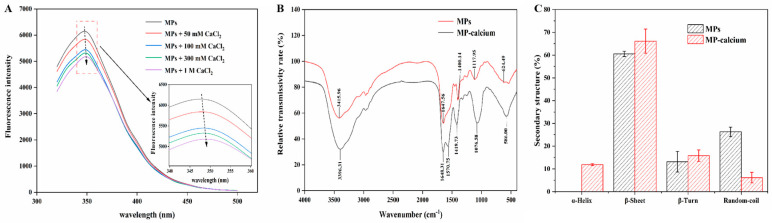
Structural characterization of MP–calcium complexes. (**A**) Fluorescence spectra of MPs with different concentrations of CaCl_2_. (**B**) FTIR spectra of MPs and MP–calcium complexes in the regions from 4000 to 400 cm^−1^. (**C**) Secondary structure content of MPs and MP–calcium complexes.

Aromatic AAs exhibit endogenous fluorescence at specific wavelengths, which can be used as references for measuring the participation of aromatic AAs in metal chelation [36]. The addition of exogenous factors such as calcium ions will lead to changes in fluorescence patterns. Therefore, the conformational changes in peptides and their interactions with small molecules, such as Ca^2+^, can be analyzed by changes in fluorescence intensity [6]. As shown in Figure 3A, the emission wavelength of MPs reached a peak at approximately 350 nm, and after binding with Ca^2+^, the fluorescence intensity of the MPs decreased. With increased CaCl_2_ concentration, the endogenous fluorescence decreased, and the maximum absorption peak of the sample was redshifted, which may result from the fluorescence quenching effect of Ca^2+^ [30]. Calcium ions caused the structure of MPs to fold, which reduced the fluorescence intensity. Furthermore, π-π stacking between MPs and calcium ions may induce MP conformational changes. The change in fluorescence intensity indicated that Ca^2+^ can be assembled with aromatic AAs through π-π stacking, causing the structure of MPs to fold.

#### 3.2.2. FTIR Spectroscopy

The FTIR spectra of MPs and MP–calcium are shown in Figure 3B. The absorption peaks in the FTIR spectra typically change as the metal ion binds to the ligand atom. After calcium ions bound with the MPs, the wavenumber of the peak (3415.96 cm^−1^) produced by N-H and O-H tensile vibrations in the MPs changed, which may be caused by the participation of amino groups in calcium binding. The wavenumber range for the amide I band was 1690–1630 cm^−1^, caused by the tensile vibration of the carbonyl group (C=O), while the amide II band was attributed to N-H deformation and C-N tensile vibration. When calcium ions bind with MPs, the amide I band is blue-shifted, and the amide II band shifts to a lower wavenumber (1570.75 cm^−1^). This phenomenon is related to the tensile vibration of the carboxyl group, indicating that -COO- changes to -COO-Ca [37]. Additionally, the peak of MPs at 1117.95 cm^−1^ may have been caused by the symmetric tensile vibration of -PO_3_, where the peak of -PO_3_ in MP–calcium shifted (1076.58 cm^−1^), indicating that phosphate groups may also participate in Ca^2+^ binding [38]. The wave numbers of the C-H and N-H bonds correspond to a range of 500–1000 cm^−1^, corresponding to a range from 624.49 to 586.00 cm^−1^, indicating that the C-H and N-H bonds were replaced by N-Ca bonds [39]. Therefore, combined with the AA analysis, the carboxyl and amino groups of Asp and Glu of the MPs may be the binding sites for calcium ions.

The secondary structures of MPs and MP–calcium complexes are shown in Figure 3C. When Ca^2+^ bound to the binding site of MPs, the β-sheet and β-turn content increased, which might be due to the carboxyl groups in the acidic AAs of MPs binding to Ca^2+^, losing their original negative charge and reducing their repulsive force, leading to folding or aggregation and MP–calcium nanocomposite formation [12,40]. Additionally, the content of ordered structures (α-helices) increased while that of disordered structures (random coils) decreased; therefore, we inferred that MPs may form a more compact secondary structure when combined with Ca^2+^ [38], and folding or aggregation may occur during binding, and the structure becomes more ordered.

### 3.3. Microstructure Analysis of MP–Calcium Complexes

#### 3.3.1. AFM

**Figure 4:**
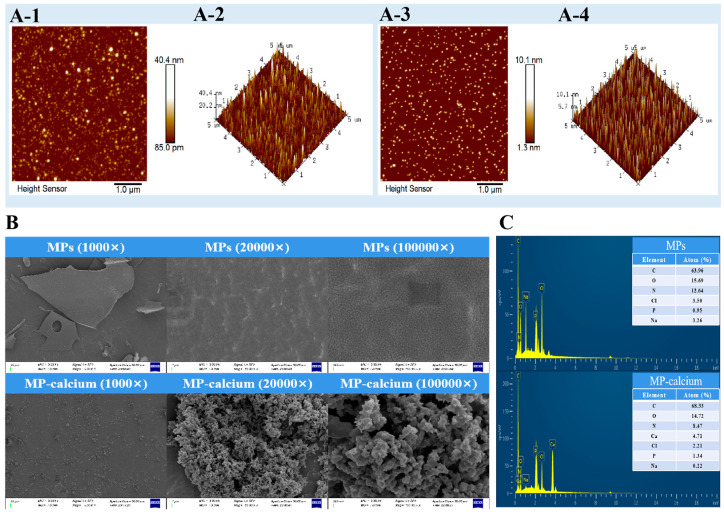
Physical characterization of MPs and MP–calcium complexes. (**A**) The typical AFM surface morphology and 3D images of MPs and MP–calcium complexes. A-1 was the surface morphology of MPs; A-2 was 3D images of MPs; A-3 was the surface morphology of MP–calcium complexes; A-4 was 3D images of MP–calcium complexes. (**B**) The microstructures of MPs and MP–calcium complexes determined via SEM. (**C**) The EDS diagram of MPs and MP–calcium complexes.

By detecting the extremely weak interatomic forces between a sample’s surface and a microforce sensor, AFM can be used to examine a sample’s surface characteristics, including its morphology, structure, and roughness [12]. The surface morphologies of the MPs and MP–calcium complexes are shown in Figure 4A-1 and Figure 4A-3, respectively, and their 3D images show their features more vividly and intuitively (Figure 4A-2,A-4). As shown in Figure 4A-1, the MPs formed a granular structure characterized by uniform distribution and dispersion. The presence of calcium ions led to an increase in the number of particles, smaller particle sizes, more concentrated distribution, and partial aggregation combined with the above analysis. This suggests that the AAs in MPs may have electrostatic interactions, hydrophobic interactions, and π-π stacking, causing the spatial structure of calcium ions and the carboxyl, amino, and phosphate groups of MPs to fold and form more uniform nanoscale particles. As the MP–calcium complexes exhibited a nanoscale structure, we speculated that MPs and calcium ions were not merely a simple combination of AA residues and calcium ions under ideal conditions but a self-assembly of multiple peptide segments and calcium ions. The formed MP–calcium complexes exhibited a nanoscale structure and were concentrated, differing from MPs.

#### 3.3.2. SEM and EDS Findings

When MPs were combined with calcium ions, the chemical bond and structure of MPs changed due to the interaction between MPs and calcium ions, resulting in morphological characteristics that differed from those of MPs. Therefore, microstructures of the MPs and MP–calcium complexes were observed using SEM, as shown in Figure 4B. The surfaces of the MPs exhibited relatively smooth lamellar structures, while the surface of the MP–calcium complexes appeared rough, with a spherical particle-folding structure. This change in microstructure may be due to electrostatic interactions, hydrophobic interactions, and π-π stacking, which disrupt the original compact structure of the MPs, consistent with the results of AFM. Additionally, the interaction of carboxyl and amino groups in the MPs combined with Ca^2+^ lead to a “bridging effect”, which can also alter their properties [41]. The elemental compositions of MPs and calcium were further studied using EDS, with energy spectra presented in Figure 4C. This energy spectrum analysis showed that the main elements in the MPs were C, N, and O, and upon binding with calcium ions, a calcium peak could be observed, indicating that calcium exists in the MP–calcium complexes. On the one hand, the increase in carbon content may be due to the self-assembly of the peptide in the process of binding with calcium ions, and the increase in peptide bonds, thus increasing the overall carbon content. On the other hand, since the phosphorylated phosphate groups may be the calcium ion binding sites, they will change the nitrogen content of the peptide after binding with calcium ions, resulting in a decrease in nitrogen content. Therefore, both SEM and EDS analyses confirm that calcium ions successfully bind with MPs to produce new compounds. They presented different morphological characteristics, which may be the result of electrostatic interactions, hydrophobic interactions, and π-π stacking.

### 3.4. Bio-Accessibility Measurement of the MP–Calcium Complexes

#### 3.4.1. Stability Analysis of MP–Calcium Complexes at Different Temperatures

**Figure 5:**
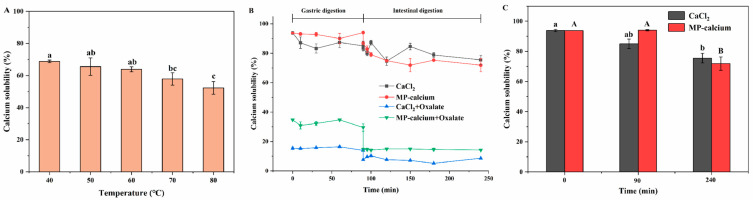
Bio-accessibility measurement of the MP–calcium complexes. (**A**) Calcium solubility of MP–calcium complexes at different temperatures. (**B**) Calcium solubility during simulated gastrointestinal digestion of MP–calcium complexes or CaCl_2_ with or without oxalate. (**C**) Calcium solubility of MP–calcium complexes after gastric and intestinal digestion. Different letters indicate significant difference among groups (*p* < 0.05).

Heat treatment is a common processing method used in most foods. This method can be used to examine MP–calcium complex stability to determine whether such complexes can be effectively applied as a calcium supplement in processed food products. The stability of MP–calcium complexes at various temperatures is shown in Figure 5A. As temperature increased, the calcium solubility of MP–calcium decreased. At 40–70 °C, high solubility was maintained, while at very high temperatures (80 °C), the calcium solubility significantly decreased. This is attributed to high temperatures causing the degradation of calcium within the MPs, significantly reducing the solubility of calcium [20]. These results indicate that the MP–calcium complexes are relatively stable over a wide temperature range and can be used as a stable calcium supplements.

#### 3.4.2. Stability Analysis of MP–Calcium Complexes under GI Digestion In Vitro

Oxalic acid is a calcium absorption inhibitor that easily forms insoluble salts with Ca^2+^ in the intestinal environment, reducing calcium absorption and utilization [24]. This study analyzed the calcium solubility of MP–calcium under simulated GI digestion using CaCl_2_ as a control. As shown in Figure 5B, the Ca^2+^ Ca^2+^ solubilities of MP–calcium and CaCl_2_ during gastrointestinal digestion remained high at 71.88% and 75.48%, respectively. As shown in Figure 5C, the solubility was high in the stomach digestion stage but decreased after entering the intestinal digestion stage, possibly due to trypsin hydrolyzing the MP–calcium, resulting in the separation of peptide and calcium, and to the alkaline pH, which facilitated the formation of Ca(OH)_2_ [21]. We also studied the Ca^2+^ solubility of MP–calcium coexisting with oxalic acid under simulated GI digestion. After adding oxalic acid to the MP–calcium and CaCl_2_ solutions, the solubility of calcium significantly decreased. From the end of gastric digestion to the initial stage of intestinal digestion, when the pH was adjusted to 7.5, the solubility of calcium significantly decreased and then stabilized after a slight increase. Moreover, after simulating gastrointestinal digestion, the calcium solubility of the oxalic acid in MP–calcium was 14.23%, significantly higher than that in CaCl_2_ (8.63%) (*p* < 0.01). These results indicate that in the simulated stomach environment, MP–calcium complexes benefited calcium transport, and in the more alkaline-simulated gut environment, MP–calcium complexes partially reduced Ca^2+^ precipitation. Furthermore, calcium in MPs can maintain its solubility under simulated GI digestion, even in the presence of oxalic acid, so that MP–calcium can be absorbed by intestinal epithelial cells to improve calcium bioavailability.

## 4. Conclusions

The present study aimed to investigate the potential utilization of MPs to enhance calcium bioavailability in vivo. To this end, we determined the best enzyme for the preparation of MPs with high calcium-binding capacity. We studied the calcium-binding capacity and changes in DH, MW, and charge distribution with enzymolysis time. Additionally, we structurally characterized MP–calcium complexes, and their GI stability was evaluated in the presence of oxalic acid utilizing an in vitro model. The results showed that the maximum calcium-binding capacity of the MPs was 120.95 mg/g when alcalase was used for 3 h, with a DH of 15.45%. Moreover, with an increase in hydrolysis time, the MW of the MPs decreased, and the negative charge increased. The combined fluorescence spectrum and FTIR results showed that the carboxyl and amino groups in Asp and Glu may be calcium-binding sites which further assemble into nanoscale spherical complexes via intermolecular interactions. The microstructure analyses confirmed that the MPs and calcium ions self-assembled to form compact, nanoscale complexes with spherical structures. Moreover, even under the influence of oxalic acid, MP–calcium maintained a certain solubility. These results support our hypothesis that MPs combined with calcium could provide a low-cost and highly bioavailable calcium supplement. However, further research ought to concentrate on the absorption mechanism of the MP–calcium complexes.

## Figures and Tables

**Table 1:** Analysis of AA composition.

AAs	MPs		MP–Calcium	
	Content (g/100 g)	Relative Content (%)	Content (g/100 g)	Relative Content (%)
Asp + Asn	6.02	8.58	9.86	14.82
Thr	2.67	4.52	3.25	4.88
Ser	2.60	3.70	3.01	4.52
Glu + Gln	8.63	12.29	14.84	22.30
Gly	3.27	4.64	3.25	4.90
Ala	3.67	5.23	2.63	3.97
Cys	0.63	0.89	0.63	0.93
Val	4.39	6.25	2.10	3.17
Met	5.42	7.72	2.60	3.94
Ile	5.42	7.72	3.89	5.86
Leu	6.52	9.28	3.50	5.29
Tyr	2.67	3.80	2.07	3.09
Phe	2.95	4.19	1.81	2.71
His	2.92	4.16	2.35	3.54
Lys	5.94	8.46	4.99	7.51
Arg	2.46	3.50	2.77	4.14
Pro	3.56	5.07	9.86	4.44
Negatively charged AAs	14.65	20.87	24.70	37.12
Positively charged AAs	11.32	16.12	10.10	15.19
Hydrophobic AAs	31.91	45.45	22.72	34.27
Essential AAs	33.80	48.14	22.14	33.36
Aromatic AAs	5.61	7.99	3.87	5.80

## Data Availability

The original contributions presented in the study are included in the article, further inquiries can be directed to the corresponding author.
